# Role of Cardiac Imaging Modalities in the Evaluation of COVID-19-Related Cardiomyopathy

**DOI:** 10.3390/diagnostics12040896

**Published:** 2022-04-04

**Authors:** Antonella Cecchetto, Stefano Nistri, Giulia Baroni, Gianpaolo Torreggiani, Patrizia Aruta, Valeria Pergola, Anna Baritussio, Marco Previtero, Chiara Palermo, Sabino Iliceto, Donato Mele

**Affiliations:** 1 Department of Cardiac Thoracic Vascular Sciences and Public Health, University of Padua Medical School, 35128 Padova, Italy; antonella.cecchetto@aopd.veneto.it (A.C.); baroni.giu@gmail.com (G.B.); gianpaolo.torreggiani@studenti.unip.it (G.T.); patrizia.aruta@aopd.veneto.it (P.A.); valeria.pergola@gmail.com (V.P.); anna.baritussio@aopd.veneto.it (A.B.); marco.previtero@aopd.veneto.it (M.P.); chiara.palermo@unipd.it (C.P.); sabino.iliceto@unipd.it (S.I.); 2 Department of Cardiology, CMSR, Altavilla Vicentina, 36077 Vicenza, Italy; stefanonistri41@gmail.com

**Keywords:** COVID-19, cardiac imaging, cardiac disease

## Abstract

Cardiac involvement has been described during the course of SARS-CoV-2 disease (COVID-19), with different manifestations. Several series have reported only increased cardiac troponin without ventricular dysfunction, others the acute development of left or right ventricular dysfunction, and others myocarditis. Ventricular dysfunction can be of varying degrees and may recover completely in some cases. Generally, conventional echocardiography is used as a first approach to evaluate cardiac dysfunction in patients with COVID-19, but, in some cases, this approach may be silent and more advanced cardiac imaging techniques, such as myocardial strain imaging or cardiac magnetic resonance, are necessary to document alterations in cardiac structure or function. In this review we sought to discuss the information provided by different cardiac imaging techniques in patients with COVID-19, both in the acute phase of the disease and after discharge from hospital, and their diagnostic and prognostic role. We also aimed at verifying whether a specific form of cardiac disease due to the SARS-CoV-2 can be identified.

## 1. Introduction

Cardiac imaging is widely required in the context of the coronavirus infective disease (COVID)-19, caused by the SARS-CoV-2, to recognize myocardial damage or dysfunction. Echocardiography is the first level technique used to perform the diagnosis of cardiac involvement and monitor infected patients, because it is easy to access and can be performed at the patient bedside. Although several studies have reported a high incidence of myocardial injury in patients with COVID-19 on the basis of troponin increase, myocardial injury associated with echocardiographic abnormalities correlates with a higher risk of death [1], suggesting an added prognostic value of echocardiography. An advantage of all cardiac imaging techniques is that they can localize myocardial damage, even if troponin increase cannot identify if myocardial injury occurs at the left or right side of the heart.

On the basis of current evidence, cardiac imaging techniques can play a fundamental role in evaluating patients with COVID-19 for both diagnosis and outcome definition. However, scientific literature grew very rapidly in this field and needs to be carefully analysed to summarize the most valuable information, especially with regard to the contribution of different imaging techniques. In this review we sought to verify: (1) the role of conventional and advanced cardiac imaging techniques, such as speckle tracking echocardiography (STE) and cardiac magnetic resonance (CMR), in the assessment of myocardial injury caused by COVID-19, both on the left (LV) and the right ventricle (RV); and, (2) whether these imaging techniques may identify a specific form of SARS-CoV-2-related cardiac disease.

## 2. Myocardial Damage in COVID-19 Patients

The prevalence of acute myocardial injury in hospitalized patients with COVID-19 is estimated to be around 20% [2,3,4,5]. There are multiple mechanisms proposed [6]: (1) direct cardiac damage mediated by stimulation of the angiotensin converting enzyme 2 (ACE2), which is expressed on myocytes and vascular endothelial cells and involved as receptor for SARS-CoV-2; (2) myocardial damage induced by hypoxia; (3) damage related to systemic inflammatory response mediated by release of cytokines, the so-called “cytokine storm”; and (4) macro and microcirculatory thrombosis, correlated to a state of hypercoagulability [7]. The damage is generally diagnosed by troponin increase [2,3,4,5] (considered significant above the 99th percentile of the upper reference limit) or the appearance of new electrocardiographic or echocardiographic abnormalities. These latter, as an expression of acute myocardial damage, should be of recent onset and contextual to troponin increase. This is not of secondary importance, as patients with COVID-19 often have cardiovascular comorbidities; therefore, electrocardiographic and echocardiographic abnormalities may be pre-existing and not related to the infection [8,9].

When clinical, laboratory and electrocardiographic suspicions of acute myocardial damage are present, conventional transthoracic echocardiography represents the first level investigation, primarily for the evaluation of biventricular function. Abnormal LV systolic function is generally detected by a decreased ejection fraction (EF) and/or changes in regional wall motion. RV function is characterized on echocardiography through a quantitative multiparametric assessment that includes fractional area change (FAC), tricuspid annular plane systolic excursion (TAPSE), and peak systolic velocity of the tricuspid annulus (peaks’ wave) at the tissue Doppler imaging (TDI) examination. If image quality is sufficient, RV-EF can be calculated by three-dimensional echocardiography.

The spectrum of cardiac manifestations in hospitalized patients with COVID-19 was first described by the group of Szekely and Lichter [10], who performed a complete echocardiographic evaluation within 24 h of admission in 100 consecutive patients, repeating the examination in cases with clinical deterioration. Thirty-two percent of the patients had a normal echocardiogram. The most common pathological finding was RV dilation and dysfunction (39% of patients), followed by LV diastolic dysfunction (16%) and LV systolic dysfunction expressed by a reduced EF (10%). Patients with troponin elevation (20%) or worse clinical condition showed no differences in LV systolic function compared to patients with normal troponin and better clinical condition, but had worse RV function. In patients with clinical deterioration (20%), the most common echocardiographic abnormality at follow-up was RV dysfunction (12 patients), followed by LV systolic and diastolic dysfunction (5 patients).

The largest collection of echocardiograms in COVID-19 patients was published by Dweck et al. [11], who collected data through a multicentre online survey. The study examined 1216 patients, of which 26% had pre-existing heart disease. Fifty-five percent of patients with impaired echocardiography were elderly patients with pre-existing heart disease, valvular disease, and history of heart failure. LV systolic dysfunction was diagnosed in 37.4% of patients, including 17% patients with mildly reduced EF, 12% with moderately reduced EF, and 7% with severely reduced EF. Among patients without pre-existing heart disease, 46% had an abnormal echocardiogram and 25% LV changes.

On the basis of the data reported above, it emerges that conventional echocardiography can recognize cardiac injury in patients with COVID-19 through identification of ventricular dysfunction. This technique, however, has a fundamental limitation: a negative examination does not exclude subclinical myocardial dysfunction, due to the limited sensitivity of the conventional echocardiographic parameters for recognition of the more subtle abnormalities.

## 3. The Left Ventricle in COVID-19 Patients

### 3.1. Types of Left Ventricular Disfunction

**Figure 1:**
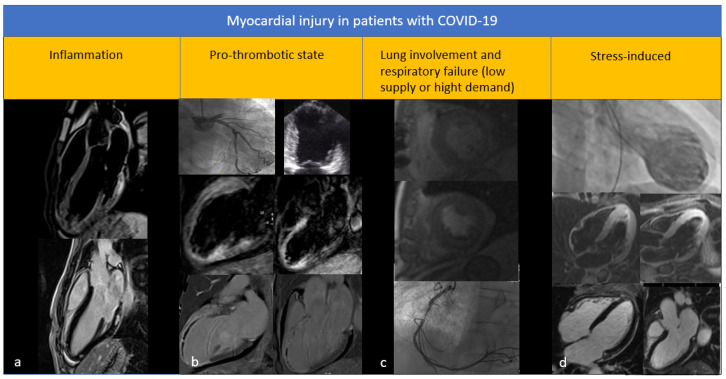
Myocardial injury in patients with COVID-19. (**a**) Case of myocarditis: CMR shows edema and fibrosis in the basal and apical infero-lateral wall of the left ventricle; (**b**) Case of myocardial infarction: coronary angiography shows occlusion of the anterior descending coronary artery; on echocardiography, alterations of the kinetics in the territory of the anterior descending coronary artery are noted and on CMR extensive areas of edema and fibrosis are described in the same location, with outcomes of microvascular damage; (**c**) Case of myocardial ischemia induced by hypoxia: stress CMR post-admission shows ischemia in the right coronary artery region, which will present a critical stenosis on coronary angiography; (**d**) Case of Tako-tsubo: ventriculography shows the typical apical ballooning appearance of the left ventricle; CMR confirms the presence of edema and the absence of fibrosis in the apical area.

Aetiology of LV dysfunction can be divided into two main categories: ischemic and non-ischemic. In the context of non-ischemic LV dysfunction, some specific pictures in COVID-19 patients can be distinguished: stress-induced ventricular dysfunction, cytokine ventricular dysfunction, and myocarditis [12]. Figure 1 shows the main types of myocardial damage observed in patients with COVID-19 infection.

(a) Stress-induced ventricular dysfunction. Stress-induced ventricular dysfunction (also known as tako-tsubo cardiomyopathy) has been observed in patients with COVID-19, the first published by Meyer et al. [13]. The trigger has not been established. However, if we consider the physical and psychological stress associated with the infection, it is not surprising that the tako-tsubo cardiomyopathy can develop in patients with COVID-19 [14]. In these cases, the clinical presentation was characterized by chest pain, associated with ECG changes (ST segment elevation and T negative waves with QT interval prolongation). The diagnosis was suspected on echocardiography, due to the typical contraction of the LV with systolic apical ballooning, and was confirmed by coronary angiography or computed tomography on the basis of absence of significant coronary lesions. Other rare patterns of the tako-tsubo cardiomyopathy are: the reverse pattern, characterized by basal akinesia/hypokinesia with apical hyperkinesia; the global pattern, with diffuse hypo/akinesia and apical sparing; and, the biventricular and focal variants [15]. A retrospective review of 169 cases performed by an American study group identified seven patients with clinical and echocardiographic features suggestive of tako-tsubo cardiomyopathy, of which three had typical echocardiographic appearance (apical ballooning), 2 reverse or mid-ventricular pattern, one biventricular pattern and one global pattern [16]. In-hospital mortality was 57%, which is very high if we consider the generally good prognosis of patients with stress cardiomyopathy. The echocardiographic elements that identified patients at higher risk were a reduced EF (≤45%), more than mild mitral regurgitation, RV dysfunction, and sinus tachycardia at the time of the examination. However, absence of coronary artery disease was not reported in this study [16].

(b) Cytokine ventricular dysfunction. In patients with COVID-19, heart failure is not uncommon, probably in response to a systemic inflammatory response, immune-mediated myocardial injury, or hypoxemia associated with progression of lung disease. In the context of COVID-19, dysregulation of the cytokine system has been recognized as a possible cause of myocardial dysfunction [6]. The hypothesis arises from severe ventricular dysfunction observed in other acute conditions characterized by cytokine-mediated responses, such as septic shock and cardiogenic shock secondary to acute myocardial infarction [17,18]. From an echocardiographic point of view, LV dysfunction due to cytokine system dysregulation does not have peculiar morphological features. It can determine a widespread impairment of myocardial function, which can be reversible. A case of severe transient LV systolic dysfunction in COVID-19 has been reported in the literature, without a typical phenotype of stress cardiomyopathy [19]. The patient’s instability required inotropic and mechanical support with extracorporeal membrane oxygenation (ECMO). Myocardial biopsy, showing only modest signs of inflammation, led to the exclusion of fulminant myocarditis, supporting the hypothesis of ventricular dysfunction secondary to cytokines. Cardiac function fully recovered in 5 days and ECMO was removed. Twelve days after weaning from ECMO, the patient suddenly developed Gram-negative pneumonia (*Pseudomonas aeruginosa* and *Klebsiella pneumoniae*) and died of septic shock within a few hours, without any further LV function impairment [19].

(c) Myocarditis. The diagnosis of myocarditis should be made following international indications also in patients with COVID-19 [20,21,22]. The 2013 position paper of the European Study Group of Myocardial and Pericardial Diseases [20] distinguishes clinically suspected myocarditis (conceivable using clinical, laboratory, and echocardiographic indicators) from biopsy-confirmed myocarditis. However, in common clinical practice, endomyocardial biopsy is not performed in absence of signs of heart failure and arrhythmias; it is not available in all hospitals and in a pandemic period it is more difficult to carry out due to both the difficulties in transporting critical patients and the risk of spreading the disease. Therefore, echocardiography remains the main cardiac imaging method to suspect this diagnosis [23]. At the present time, there are no biopsy demonstrations of myocarditis directly due to SARS-CoV-2 in humans [24]. The lack of biopsy detection of viral material in cardiomyocytes agrees with other biopsy and autopsy findings, in which viral particles were found in cardiac macrophages but not in cardiomyocytes and endothelial cells [25]. The presence of viral particles in cardiac macrophages has been interpreted as the result of a viraemic phase or the migration of infected macrophages from the lungs into extrapulmonary tissues. Among the factors that increase the diagnostic uncertainty of myocarditis, the indiscriminate or unspecified use of the term myocarditis in some scientific works [26] and the non-homogeneity of the criteria used for this diagnosis have been recognized. Acute myocarditis, therefore, remains a possibility described in the context of coronavirus infections. The cases of clinically suspected myocarditis, published so far in reference to the current SARS-CoV-2 pandemic, had a variety of presentations. LV-EF could be normal or mildly, moderately, or severely reduced [24]. Some cases had a prevalent pericardial expression and pericardial effusion with severe tamponade [24]. Most cases of clinically suspected myocarditis in COVID-19 overcame the acute phase, with EF recovery within normal limits after treatment [24]. However, the evolution of some cases remains unknown.

### 3.2. Findings from Speckle Tracking Echocardiography

Although LV-EF is the most widely used measure of LV systolic function, global longitudinal strain (GLS) obtained by two-dimensional STE is considered a more accurate method for early detection of subclinical LV dysfunction. This method evaluates contraction of the LV longitudinal myocardial fibers, located especially in the subendocardium, which is most vulnerable to ischemia or injury. Therefore, GLS may identify myocardial damage at an early stage and prior to reduction of LV-EF.

Van den Heuvel et al. [27] found systolic dysfunction in 27% of 51 consecutive patients; 50% of patients with ventricular dysfunction had a GLS reduction. Baycan et al. [28] in 100 patients with normal EF found a reduction in GLS in comparison with a control group, regardless of the severity of COVID-19. It has also been reported that LV-GLS provides additional prognostic information. In patients with COVID-19, LV strain reduced regardless of EF values and was a predictor of mortality. Chang et al. [29] proposed absolute GLS cut-off values of <13% as the best marker for mortality in patients with septic shock in the intensive care. Correlations between GLS and clinical and laboratory evaluations in patients with COVID-19 are reported in Table 1.

A frequent localization of strain reduction at the LV basal segments has been reported, suggesting an area of the myocardium more susceptible to virus-induced damage [30,31]. However, these cases had higher body mass index and were more frequently black, hypertensive, and diabetic compared with controls, thus evidencing that other factors could be implicated in the genesis of the LV basal dysfunction. It is possible that the basal injury pattern might reflect the susceptibility of certain myocardial regions to inflammatory or systemic stressors. Another hypothesis involves the viral receptor, ACE2. This is highly expressed in fat and epicardial adipose tissue, which is more prominent at the atrioventricular groove and lateral LV wall, closer to the basal segments. Loss of ACE2 has been shown to result in heart failure with preserved EF, mediated in part by epicardial adipose tissue inflammation. Thus, binding of severe acute respiratory syndrome coronavirus to ACE2 might occur more prominently in areas of high epicardial adipose tissue, such as the basal LV, and cause subclinical dysfunction via inflammatory downstream effects, perhaps more readily in overweight and obese patients [30].

The significance of strain reduction is still debated in patients with COVID-19 and the relationship with high troponin levels remains unknown. Ozerl et al. [32] used LV-GLS to evaluate whether there is subclinical myocardial deformation after COVID-19 infection. Two-dimensional STE was performed within 1 month after hospitalization for COVID-19 infection. The patients were divided into two groups according to their troponin levels during hospitalization, with and without myocardial injury. An abnormal LV-GLS value (>−18%) was observed in 28 patients (37.8%). Of these patients, 57.1% were in the group with increased troponin (myocardial injury) and 26.1% in the group without. The fact that one quarter of patients had a LV-GLS reduction in the group without myocardial injury might suggest that all patients should be evaluated during the follow-up period after discharge, regardless of troponin levels during hospitalization. Balaban Kocas et al. [33] investigated the relationship between troponin levels and LV-GLS values in patients with COVID-19. A total of 38 patients diagnosed with COVID-19 pneumonia were enrolled and underwent echocardiography examination within the first week of hospital admission. Patients were divided into two groups according to their troponin levels. Frequency of hypertension, diabetes mellitus, and smoking were similar among groups. Despite troponin increase was highly related to in-hospital adverse events, no relationship was found between troponin increase and LV-GLS values of COVID-19 patients. Baykiz et al. [34] recruited 75 patients from the COVID-19 outpatient clinic for their follow-up visits at a median of 6 months after discharge. Patients were classified into groups according to pneumonia severity. LV-GLS values after discharge were significantly lower in the group with severe pneumonia than in those without pulmonary involvement. The authors hypothesized that patients of the first group may benefit from close monitoring of long-term outcomes such as heart failure and LV dysfunction. Bathia et al. [35] found abnormal values of GLS in 91% of 96 patients hospitalized with COVID-19. There was no difference in EF or GLS when stratified by symptoms or need for intensive care. There was no correlation between strain measurements and cardiovascular biomarkers. When stratified by cardiovascular disease, both groups had abnormal GLS, but presence of cardiovascular disease was associated with worse GLS. Patients who died had stable or worsening GLS, while those who survived to discharge home showed improved GLS.

Taken together, all these findings indicate that patients with SARS-CoV-2 infection have subclinical LV systolic dysfunction not adequately captured by traditional echocardiographic parameters [36,37]. However, the clinical impact of these findings requires to be clarified with further investigations. A recent method introduced in echocardiography is the determination of myocardial work, based on longitudinal strain obtained using 2D-STE. With this method, myocardial strain is indexed by systolic blood pressure, in order to obtain a load-independent measure of systolic function. In patients with COVID-19, reduced myocardial work efficiency has been associated with increased mortality and may represent an early marker of LV systolic dysfunction [38,39].

**Table 1:** Studies that assessed the correlation between LV-GLS and clinical and laboratory evaluations during COVID-19 pneumonia.

Study	N Patients	Time to Echo from Admission	GLS Abnormal Value (% of Patients)	Stratification by Comorbidity	Correlation with		
					Tn	CPR	PS
Kocas et al. [33]	38	1 week	>−18% (28.9%)	+	-	-	NK
Ozer et al. [32]	28	1 month	>−18% (37.8%)	+	+	+	NK
Baykiz et al. [34]	75	6 months	>−16%	+	-	+	+
Li et al. [36]	218	average of 24 days	>−21% (83%)	+	+	+	-
Hayama et al. [37]	209	average of 56 days	>−20% (29.7%)	+	+	NK	NK
Bathia et al. [35]	67	1 week	>−18% (91%)	+	-	NK	-
Baycan et al. [28]	100	1 day	NK	+	+	-	+

Tn = troponin, CPR = C-protein reactive, PS = pneumonia severity; NK = not known.

### 3.3. Findings from Cardiac Magnetic Resonance

Once LV dysfunction is recognized, aetiology should be determined. To this purpose, second level cardiac imaging techniques, such as CMR, computed tomography, or coronary angiography, should be considered to determine the cause of troponin increase and/or mechanical dysfunction, depending on the clinical scenario. CMR is useful in characterizing myocardial tissue and confirm the presence, type, and extent of myocardial damage. In inflammatory damage, late gadolinium enhancement (LGE) presents a morphological pattern with a predominantly intramural or epicardial distribution, associated with signs of inflammation visible in the T2-weighted sequences. In ischemic disease, the LGE is localized at the subendocardium and can extend into the thickness of the myocardial wall, also reaching transmurality. There is also a third possibility, represented by the tako-tsubo cardiomyopathy, in which functional abnormalities, such as the apical ballooning, are found in the presence of edema in the akinetic segments, but without signs of LGE. All these pathological pictures have been associated with COVID-19.

A multicenter CMR study, conducted in six hospitals in London in patients who recovered after COVID-19 with troponin elevation, showed LGE abnormalities in about 54% of cases, with three characteristic damage patterns: non-ischemic (26% of cases), ischemic (22% of cases), and non-specific non-ischemic scar (5% of cases) [40]. A dual component, ischemic and non-ischemic, was observed in 6% of cases. This might indicate that several factors are involved in the genesis of myocardial damage, such as myocarditis, myocardial infarction (type 1 or 2), and myocardial ischemia. Among patients with myocarditis pattern, one third of them showed evidence of ongoing active myocardial inflammation at the early post-infection stage. These patients should be probably followed-up carefully. In fact, inflammation and LGE may play a role in the pathophysiology of dilated cardiomyopathy [41]. Studies are also needed to clarify whether there is a long-term presence of scar, which is associated with adverse cardiac events following myocarditis [42]. Among patients with ischemic pattern, most had at least one risk factor for cardiovascular disease, thus it is likely that a large number of them had an underlying coronary artery disease that was unmasked by the systemic infection (type 2 myocardial infarction). An alternative hypothesis is that a proportion of myocardial infarctions in these patients could be the result of a pro-thrombotic state occurring during the acute infection. Regardless of the mechanism underlying ischemic myocardial injury, these patients are important to identify, as they could benefit from prognostic medical therapy and be considered for coronary intervention in the presence of significant obstructive disease [40]. On the basis of this study, myocardial injury appears to be common in hospitalized patients with COVID-19 and not exclusive to those with acute coronary syndromes or pulmonary emboli.

In a single-center, single time point convalescent study, a CMR scan was offered to patients discharged with a COVID-19 diagnosis and with myocardial injury indicated by elevated troponin [43]. Among the 51 patients referred for CMR, 22 had ≥1 identifiable causes of troponin elevation (6 had acute coronary syndromes, 12 pulmonary emboli) or known cardiac pathology (7 had a history of ischemic heart disease) or both. The remaining 29 patients had unexplained myocardial injury and no cause for previous myocardial scarring and 19 (66%) of them underwent additional adenosine stress perfusion. With the use of the LGE technique and (where possible) stress perfusion imaging, 20 patients (69%) had an identifiable mechanism of myocardial injury, classified as non-ischemic heart disease-related (11 patients, 38%), ischemic heart disease-related (5, 17%), or dual ischemic and non-ischemic pathology (4, 14%). A non-ischemic cause of elevated troponin was conferred by the presence of non-infarct pattern LGE (not corresponding to a coronary territory and sparing the endocardium).

A recent study reports the first series of patients with COVID-19 consecutively referred for CMR for suspected myocarditis in four Italian university hospitals [44]. High-sensitivity troponin concentrations were elevated. Obstructive coronary artery disease was excluded in nine patients. All patients underwent CMR within 1 week from troponin rise and onset of cardiac symptoms. Two patients had severe depression of systolic function with apical ballooning, apical edema, and absent LGE, suggesting tako-tsubo cardiomyopathy. Five patients had preserved EF and three patients had mildly reduced EF. In all cases, CMR showed diffuse intense myocardial edema, with increased myocardial-to-skeletal muscle intensity ratio on STIR images, increased native-T1 mapping, and increased T2 mapping. LGE images were completely negative in five of eight patients. In the remaining three patients, a few thin and shadowed subepicardial striae of LGE were detectable in the lateral wall. Based on updated 2018 Lake Louise criteria, CMR findings, including T1 and T2 myocardial markers, resulted in diagnosis of acute myocarditis in all eight patients. The absence or very minimal amount of LGE observed in these patients agrees with the few histological results published to date, reporting limited or absent myocyte necrosis, and may suggest an indirect mechanism causing myocardial inflammation. 

Other papers documented that, in patients with early-stage COVID-19, myocardial edema and functional abnormalities are a frequent finding, while irreversible regional injury such as necrosis may be infrequent. Chen et al. [45] reported that, among 25 COVID-19 patients, 14 (56%) displayed hyperintensity in T2-STIR and 1 (4%) patient displayed positive LGE. Most of the T2-STIR hyperintensity was mainly distributed in the transmural myocardium, while only one patient displayed hyperintensity on T2-STIR consistent with LGE in the subepicardium of the inferior and inferior-lateral wall in the basal and mid-portions of the LV. To explain this phenomenon, the authors hypothesized that, in the absence of LGE in most cases in the early stages of the disease, edema may reflect reversible myocardial injury. The prevalence of edema over fibrosis in patients with and without cardiac symptoms and elevated serologic markers of myocardial injury during the whole course of COVID-19 has been considered in several studies. In particular, mapping abnormalities were more prevalent than ventricular dysfunction. This further strengthens the value for mapping techniques as a sensitive tool for detecting early myocardial involvement in COVID-19 [46,47].

Interesting insights come from the study of athletes with COVID-19. Clark et al. conducted a CMR study within 1 month of infection on athletes, with normal electrocardiograms, troponin, and echocardiograms with strain, compared to healthy athletes and healthy controls [48]. Most standard CMR parameters were similar between COVID-19 positive athletes and athletic controls. Focal LGE isolated to the inferoseptal RV insertion was present in 22% of COVID-19 positive athletes, compared with an identical LGE pattern in 24% of athletic controls. Mild segmental increases in T1, T2, or extracellular volume were found in 39% of COVID-19 positive athletes, 13% of athletic controls, and 8% of healthy controls compared with their laboratory-specific normative values. This study suggests that the prevalence of myocardial inflammation or fibrosis after an asymptomatic or mild course of ambulatory COVID-19 among competitive athletes is modest, but would be missed by ECG, troponin, and strain echocardiography.

Looking at all studies published so far, a number of patients had normal CMR despite cardiac symptoms and biomarker levels. This can have two possible explanations. Considering the fact that normal CMR was more commonly seen in case series with a higher gap between symptoms and time of acquisition, the most likely reason is that patients may have had myocarditis, but were imaged later in the course of the disease, when edema had already resolved. Another reason could be that symptoms were because of residual pulmonary involvement rather than the cardiac [49].

Follow-up studies in COVID-19 survivors found persistent symptoms (fatigue, dyspnoea, muscle pain etc.), impaired pulmonary function, and abnormal chest CT images. However, whether COVID-19 has a continuous influence on the cardiovascular system in late convalescence is unknown. Wang et al. [50] aimed to evaluate mid-term cardiac sequelae in recovered COVID-19 patients by CMR. A total of 44 recovered COVID-19 patients and 31 healthy controls were prospectively recruited and underwent CMR examination after a 3-month follow-up period. Among all 44 recovered patients, normal ECGs were revealed in 43 patients. ST segment elevation in leads II, III, AVF, and prolonged PR interval during initial diagnosis was demonstrated in one patient. LGE was found in 13 (30%) of COVID-19 patients. Troponin was significantly different in the LGE group and the non-LGE group. All LGE lesions were located in the mid myocardium and/or sub-epicardium, with a scattered distribution. Among a total of 208 myocardial segments in 13 patients, most LGE lesions were located at the inferior and inferior-lateral segments at the base and mid-level of the LV. The median of LGE/myocardium ratio was 1.7%. Further analysis showed that LGE-positive patients had significantly decreased LV peak global circumferential strain (GCS), RV peak GCS, and RV peak GLS as compared to non-LGE patients, while no difference was found between the non-LGE patients and healthy controls. In conclusion, this study shows that CMR can monitor the COVID-19-induced myocarditis progression. In a cohort of 34 patients, Fu et al. [51] screened for cardiac sequelae in the late convalescence using CMR. They found that cardiac involvement, including RV systolic dysfunction, segmental LV deformation decrease, myocardial edema, and fibrosis were not uncommon even after 6 months of recovery. Abnormal findings in COVID-19 survivors after 110 days and 6 months imply continuous inflammation, which may be the reason for the lasting cardiac involvement in these patients. They also identified that elevated LDH, the presence of cardiac abnormalities at admission, and the severity of COVID-19 were risk factors for cardiac sequelae in COVID-19 survivors in the late convalescent stage. Interesting, none of these 34 patients reported cardiovascular-related symptoms or signs during follow-up.

If severity of pneumonia can play a role in the cardiac follow-up is another question to answer. Li et al. [52] faced this issue in a prospective observational cohort study that enrolled 40 participants with moderate or severe pneumonia and no cardiovascular medical history, without cardiac symptoms, with normal ECG, normal serological cardiac enzyme levels, and discharged > 90 days. Cardiac function, native T1, extracellular volume, and 2D-strain were quantitatively evaluated and compared with controls. Only one (3%) participant had positive LGE located at the LV mid inferior wall. Global extracellular volume values were elevated in both participants recovered from COVID-19 with moderate or severe pneumonia, compared to the healthy controls. The LV 2D-GLS was reduced in both groups of participants compared to healthy control group. CMR myocardial tissue and strain imaging parameters suggest that a proportion of participants that recovered from COVID-19 had subclinical myocardial abnormalities detectable months after recovery.

In summary, CMR is a fundamental tool in the differential diagnosis of patients with COVID-19 myocardial injury. The characterization of myocardial tissue allows to quantify myocardial damage and to clarify its etiological nature. This differentiation is fundamental not only for understanding the pathophysiological mechanisms associated with myocardial damage induced by COVID-19, but also for defining the most effective therapeutic treatment. CMR can also monitor the progression of COVID-19-induced myocardial injury. At present, however, there no established protocols in this regard.

## 4. The Right Ventricle in COVID-19 Patients

### 4.1. Characteristics and Importance of Right Ventricular Dysfunction

Isolated RV dysfunction in patients with COVID-19 may suggest acute pulmonary hypertension, right myocardial infarction, or focal myocarditis. Given the high incidence of acute respiratory distress syndrome (ARDS) requiring mechanical ventilation and the state of hypercoagulability responsible to the development of deep vein thrombosis and pulmonary embolism, lung parenchyma and pulmonary microcirculation damage may contribute to RV dilation and dysfunction. In addition, positive end-expiratory pressure, common ventilatory support, can acutely worsen RV function, and this persistent dysfunction correlates with an adverse prognosis [53].

Data on RV involvement in COVID-19 have been published. The group of Szekely and Lichter [10], who first conducted the above-mentioned echocardiographic study within 24 h of patients’ admission, described RV dilation in 40% of patients, with or without dysfunction. The pulmonary acceleration time (AT) was reduced, demonstrating an increase in pulmonary vascular resistance, and FAC and the peaks’ wave at TDI were lower than in normal values, demonstrating a reduced RV function. The reduction in AT, which suggests a RV afterload increase, assumed a prognostic value, being associated with more complex clinical pictures. Progressive clinical deterioration observed in 20% of patients and troponin increase were related to RV dilatation and dysfunction. Other investigators provided further information. Lazzeri et al. [54] demonstrated, in a population of 42 patients with moderate to severe ARDS, that troponin release was related to RV dysfunction. Argulian et al. [55] conducted an echocardiographic study on 105 consecutive hospitalized patients, of which 30% had mechanical ventilatory support at the time of the echocardiographic examination. RV dilation was observed in 31% of patients and on multivariate analysis, RV dilation was the only variable significantly associated with mortality. To evaluate the prevalence and prognostic value of pulmonary hypertension and RV dysfunction in patients admitted to non-intensive care units, Pagnesi et al. [56] conducted an observational echocardiographic study on 200 patients. The prevalence of pulmonary hypertension (PAPs ≥ 35 mmHg) was 12% and that of RV dysfunction (TAPSE < 17 mm or s-wave < 9.5 cm/s) 14.5%. Patients with pulmonary hypertension were older and with more comorbidities and with signs of more severe pulmonary impairment, in radiological, laboratory, and oxygen saturation terms. Patients with RV dysfunction, on the other hand, had more comorbidities but no evidence of more severe lung disease. Furthermore, patients with pulmonary hypertension, unlike those with RV dysfunction, were associated with a higher rate of in-hospital mortality and ICU transfer. On the basis of these findings, it is imperative for the clinician to pay attention to RV function in hospitalized patients, especially in patients with severe pneumonia, ARDS, and on ventilation.

### 4.2. Information from Speckle Tracking Echocardiography

In a study by Li et al. [57], 120 consecutive patients with COVID-19 underwent echocardiography to study RV systolic function, using both conventional parameters (such as FAC, TAPSE and peak s’ wave) and the RV free wall longitudinal strain (FWLS) by 2D-STE. Patients with greater impairment of RV longitudinal deformation (strain cut-off value of −23%) had increased heart rate; elevated CRP and D-dimer values; increased incidence of acute myocardial injury, ARDS, and deep vein thrombosis; higher mortality; and received high flow oxygen and mechanical ventilation. Interestingly, RV-FWLS predicted mortality more accurately than FAC, becoming an important echocardiographic marker of patients at-risk. Most notably, patients were included without categorizing them according to their comorbid status. Furthermore, detection of RV longitudinal strain as an independent predictor of mortality might be due to the enrolment of a relatively high number of patients with significant comorbidities. Gibson et al. [58] determined the RV-FWLS in 32 patients receiving mechanical ventilation for COVID-19-associated respiratory failure. They proved that abnormal RV-FWLS was present in the majority (66%) of patients and, unexpectedly, was associated with favourable lung mechanics (i.e., compliance) and lower airway pressures, suggesting that RV-FWLS in this population may not be attributable to alveolar collapse or distension during positive pressure ventilation. RV-FWLS correlated negatively with age and with serum troponin. Patients with abnormal RV-FWLS did not exhibit worse oxygenation, hypercarbia, or acidosis, and, consistent with these findings, did not have radiologic evidence of more severe lung disease to account for the RV impairment. The authors considered two alternative mechanisms to explain impaired RV function: pulmonary vascular abnormalities, such as micro or macro thromboembolic phenomena that can increase RV afterload, and direct myocardial injury causing impaired contractility. Direct myocardial damage could occur in severe COVID-19 either from inflammation or viral entry into cardiomyocytes, which, however, has not been demonstrated so far. Lastly, they found that abnormal RV-FWLS was associated with markers of reduced LV systolic and diastolic function. LV dysfunction in previously normal hearts may be seen during acute pulmonary hypertension as a consequence of ventricular interdependence. Alternatively, RV-FWLS may provide an early indication of global cardiac impairment in COVID-19 patients, as factors causing direct injury to the RV would also damage the LV.

Studies evaluating RV function during follow-up of COVID-19 patients have been published using 2D-STE. Günay et al. [59] enrolled 51 patients with COVID-19 (29 with severe and 22 with moderate pneumonia) and 32 healthy volunteers. They showed that RV dysfunction continued in the first month after discharge. RV-GLS strain and RV-FWLS were lesser in COVID-19 patients than in the control group. Another study confirmed subclinical dysfunction of RV by 2D-STE in hospitalized patients in relation to the severity of pneumonia after recovery from COVID-19 [60]. The median follow-up duration was 4 months. After recovery from COVID-19, echocardiography was performed in 79 consecutive patients. According to the recovery at home vs. hospital, patients were divided into two groups: home recovery (*n* = 43) and hospital recovery (*n* = 36). Comparisons were made with age, sex, and risk factor-matched control group (*n* = 41). In patients recovered from hospital, RV-GLS and RV-FWLS were impaired compared to the control group. In subgroup analysis, RV-FWLS was impaired in patients with severe pneumonia compared to mild-moderate pneumonia, without pneumonia and control groups. A significant correlation was detected between serum CRP level at hospital admission and both RV-GLS and RV-FWLS. Age, male gender, pneumonia on computed tomography, and need for steroid in treatment were identified as independent predictors of impaired RV-FWLS (>−18%) via multivariate analysis.

## 5. Conclusions

**Figure 2:**
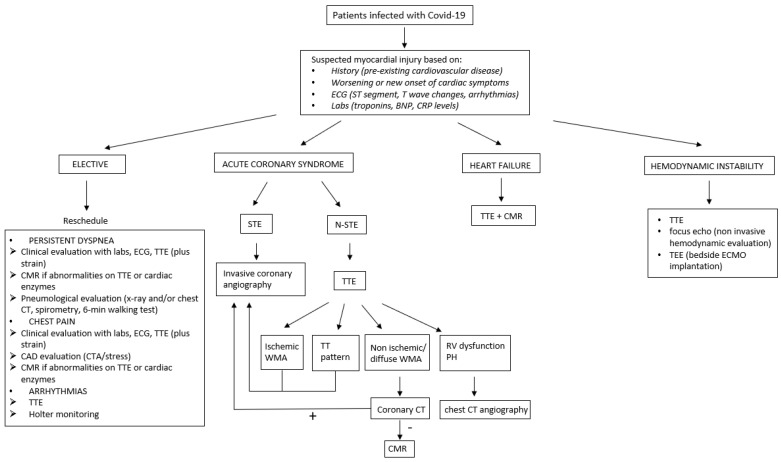
Our diagnostic approach in patients with COVID-19 and suspected myocardial damage. BNP = brain natriuretic peptide; CRP = C-protein reactive; TTE = transthoracic echo; CMR = cardiac magnetic resonance; CT = computer tomography; STE= ST elevation; N-STE = non ST elevation; WMA = wall motion abnormalities; TT = tako-tsubo; RV = right ventricle; PH = pulmonary hypertension; TEE = transesofageal echo; ECMO = extra corporeal membrane oxygenation.

Cardiac injury has been described in patients with COVID-19. Left, right, and bi-ventricular dysfunction have been reported in the acute phase of the disease, not necessarily related to an ischemic origin. Typically, ventricular dysfunction is recognized using echocardiography, but the etiological diagnosis of the dysfunction cannot usually be made based only on this diagnostic method. CMR plays a fundamental role in this regard. Figure 2 schematically shows our diagnostic approach in patients with COVID-19 and suspected myocardial damage. Advanced cardiac imaging techniques, especially 2D-STE and CMR, are essential to identify subclinical forms of myocardial damage in COVID-19 patients. This is important especially in recognizing patients who may benefit from a follow-up in the next future. In particular, those who were very ill (admitted to hospital, with severe pneumonia, having a positive troponin, elevated CPR) or with evidence of residual inflammation on early convalescent CMR may be important groups to target, because inflammation may play a role in the pathophysiology of dilated cardiomyopathy. Unfortunately, there are not established protocols on how to conduct a follow-up study using cardiac imaging techniques in patients who recovered from COVID-19. Assessment of progression of cardiac injury requires that the same cardiac imaging technique was applied during the acute phase. Finally, it is evident from current literature that there are multiple types of cardiac involvement in COVID-19 patients, probably because multiple mechanisms of myocardial injury can take place in this disease. The information available so far does not allow to define a unique form of SARS-CoV-2 cardiomyopathy.
